# Seeing through the smoke: The effects of wildfire fine particulate matter (PM_2.5_) exposure on standing and lying behavior in Holstein heifer calves

**DOI:** 10.3168/jdsc.2023-0503

**Published:** 2024-03-29

**Authors:** A. Pace, K.M. Mirkin, P. Rezamand, A.L. Skibiel

**Affiliations:** Department of Animal, Veterinary and Food Sciences, University of Idaho, Moscow, ID 83844

## Abstract

Wildfires are burning more acres annually, contributing to air pollution across the United States. Air pollutants, such as particulate matter (PM_2.5_), have health implications for humans and animals, and are known to alter behavior in several species, but effects of wildfire PM_2.5_ on dairy calf behavior are unknown. The present study aimed to understand how dairy calf standing and lying behavior is affected by wildfire PM_2.5_. Holstein heifer calves (n = 13) were monitored for the first 90 d of life, concurrent with the 2022 wildfire season. Hourly PM_2.5_ concentrations and meteorological conditions, which were used to calculate temperature-humidity index (THI), were recorded. Wildfire and wind trajectory mapping was used to determine the contribution of wildfires to spikes in PM_2.5_. Calf activity data were recorded every minute using accelerometers and analyzed as total hourly and daily standing and lying times, standing and lying bouts, and duration of bouts. Additionally, the responses of calves to the initial 24-h period of each of 2 separate exposures to wildfire smoke were assessed. Wildfire PM_2.5_ exposure was associated with reduced daily standing time and bout duration, increased daily total lying time, and increased, albeit not significantly, daily standing bouts. Percent of time standing hourly was increased, whereas percent of time lying hourly was decreased by wildfire PM_2.5_. The initial 24 h of each smoke exposure was characterized by decreased standing and increased lying time, but there was a greater change in behavior during the first event compared with the second event. These results indicate that exposure to wildfire PM_2.5_ induces a behavioral response, which may diminish with repeated exposures. Future research should aim to understand the health and welfare implications of the behavioral responses to wildfire PM_2.5_.

## Highlights

• Climate change has contributed to greater wildfire activity, resulting in PM_2.5_ pollution.
• Calves exposed to wildfire PM_2.5_ exhibit alterations in metabolic and inflammatory markers.
• Wildfire PM_2.5_ causes changes in standing and lying activity.
• Behavior changes may be interconnected with the health and metabolic effects of wildfire PM_2.5_.

## Graphical Abstract

**Figure:**
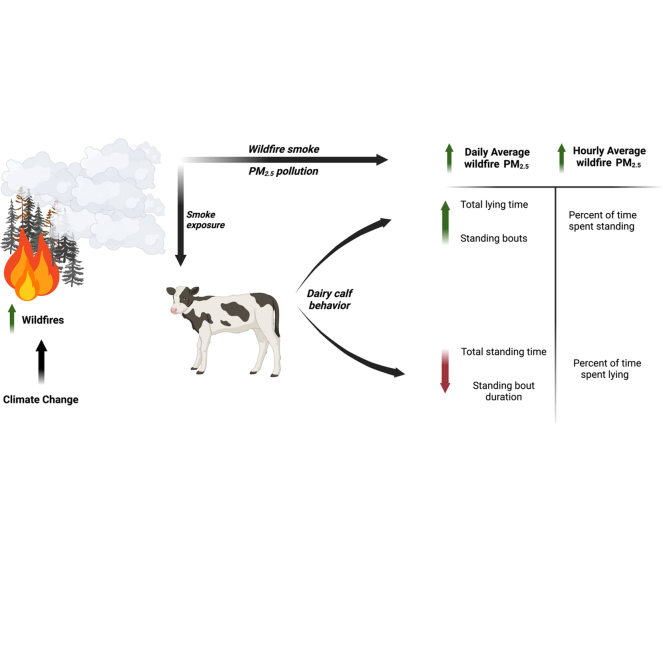
**Summary** Wildfires are increasing with climate change. Wildfires produce smoke that contains hazardous emissions, such as particulate matter (PM_2.5_), that harm human and animal health and alter behavior in nondomesticated species. We have previously found that calves exposed to wildfire PM_2.5_ experience altered metabolism and inflammatory markers, but the effects of wildfire PM_2.5_ inhalation on calf behavior are unknown. In this study, heifer calf behavior was evaluated before and during exposure to 2 natural wildfire smoke events, resulting in elevated PM_2.5_ concentrations. When exposed to wildfire PM_2.5_, calves spent more time lying and less time standing daily, but the opposite effect was observed during hourly fluctuations in wildfire PM2.5. Calves responded to a greater extent in the initial 24 hours of the first wildfire event compared with the second event. Further research is needed to evaluate the possible interconnections between smoke-induced behavioral responses and physiological responses, and how these may affect calf health and welfare.

In 2022, more than 68,000 wildfires occurred in the United States, surpassing the 5- and 10-yr national averages (NICC, 2022). Wildfires produce smoke, causing unhealthy air quality across large geographical areas as the combustion emissions disperse (Shrestha et al., 2022). Thus, individuals that reside far from the origination of fires can be at risk of exposure to fine particulate matter (**PM_2.5_**), a pollutant that is hazardous to human cardiovascular and respiratory health (Aguilera et al., 2021). Wildfire smoke exposure also adversely affects cattle production and physiology (Anderson et al., 2022; Pace et al., 2023). This is concerning for the dairy industry because half of the top 10 states in dairy production are also in the top 10 states with the most acres burned by fires annually (NICC, 2022; USDA National Agricultural Statistics Service, 2022).

Animals exhibit an array of responses when exposed to wildfire smoke, which have implications for health, metabolism, respiratory function, and ontogeny (Venn-Watson et al., 2013; Black et al., 2017). These responses are thought to be interconnected with behavioral responses to wildfire smoke exposure. For example, orangutans spend more time resting and less time locomoting, concurrent with changes in energy metabolism, during and following exposure to smoke (Erb et al., 2018). Torpor, a physiological state adopted by some animals that is characterized by reduction in metabolism and body temperature, is reduced after exposure to smoke in yellow-footed antechinus (Stawski et al., 2017), suggesting a relationship between energy allocation and air quality.

Exposure to wildfire PM_2.5_ alters vital signs, such as body temperature, respiration rates, and heart rates, as well as indicators of health, inflammation, and metabolism in preweaning Holstein heifers (Pace et al., 2023). However, behavioral responses to smoke exposure have been sparsely documented, particularly in domesticated species. The effects of wildfire smoke exposure on dairy calf behavior, an indicator of health and welfare and a valuable metric for dairy producers, has yet to be elucidated. As behavioral cues can indicate underlying health issues, knowledge about behavioral effects from wildfire smoke may provide insight into calf susceptibility to poor health outcomes following wildfire smoke exposure. The present study aimed to describe the activity patterns of calves, indicated by standing and lying behavior, as a result of exposure to wildfire PM_2.5_.

Approval for animal procedures was received from the University of Idaho Institutional Animal Care and Use Committee (protocol IACUC-2022–29). Calf health was monitored by both farm and research personnel. Treatments decisions were made by farm staff; no calves were diagnosed with or treated for any illnesses.

Holstein heifer calves (n = 14) were monitored from birth (early July 2022) to 90 d (October 2022) at the University of Idaho Dairy Center, which coincided with the Pacific Northwest wildfire season. Based on power analysis, a sample size of 9 was determined to be necessary to achieve statistical power of 0.9, with an estimated effect size of 0.2 and α level set to 0.05. All heifer calves from the summer calving cohort on farm were included in the study. Calves were observed before, during, and following 2 wildfire events, and each calf served as its own control. Within 4 h of birth, calves were fed 3.8 L of pasteurized colostrum and an additional 1.9 L within 12 h of birth. Calves received 2.8 L of raw waste milk 2 times per day until 53 d of age. From 53 to 60 d of age, calves were fed 2.8 L of waste milk 1 time per day and were weaned at 60 d of age as a single group based on average age of the group. Calves had ad libitum access to water and grain for the duration of the study, and ad libitum access to alfalfa starting at 45 d of age. Calves were housed individually in hutches with wood shavings until 63 d of age, and subsequently moved to group housing bedded with straw. Calf hutches and the group pen were located in a partially enclosed barn with natural ventilation, and therefore, calves were exposed to external air conditions, including poor air quality from wildfires.

Hourly PM_2.5_ (**hPM_2.5_**) concentrations were obtained from the Idaho Department of Environmental Quality (**DEQ**) monitoring station 5.7 km from the farm, from which daily PM_2.5_ averages were calculated. Additionally, hourly temperature and relative humidity data from the same station were used to calculate hourly and daily average temperature-humidity index (**hTHI** and **THI**, respectively). Further information on PM_2.5_ and meteorological measurements can be found in Idaho DEQ (2021). Given the topography of the area, PM_2.5_ and meteorological conditions recorded by the monitoring station reflect those on farm (Pace et al., 2023). The THI and hTHI were calculated using the following equation (Dikmen et al., 2008):

THI = [1.8 × temperature (°C) + 32] − {[0.55 − 0.0055 × relative humidity (%)] × [1.8 × temperature (°C) − 26]}.

Parameters previously established by Anderson et al. (2022) and Pace et al. (2023) were used to determine whether calves were exposed to wildfire PM_2.5_. The parameters include (1) daily PM_2.5_ concentrations (24 h averages) are above 35 µg/m^3^, following guidelines for the PM_2.5_ exposure threshold for humans (EPA, 2019), and (2) local PM_2.5_ spikes originated from active wildfires determined using National Oceanic and Atmospheric Administration (**NOAA**) HYSPLIT atmospheric transport and dispersion modeling (Draxler and Hess, 1997; Draxler, 1999; Stein et al., 2015). With this tool, air mass trajectories were tracked backward over 72 h at 50, 100, and 150 m of atmospheric height. Therefore, the air mass origination and PM_2.5_ movement could be tracked back to active wildfires.

At 7 d of age, accelerometers (Hobo Pendant G Accelerometer Data Logger, Onset Computer Corporation, Pocasset, MA) were placed on the inside of the left hind leg above the fetlock of each calf, with padding placed between the logger and skin for comfort, and secured with flexible veterinary bandages. Using 3-point axes, loggers tracked postural changes over time, which were recorded every minute. Data were offloaded from the loggers weekly, and loggers were replaced immediately afterward, allowing for continuous measurement of data over the course of the study. Calf logger data were converted from axis data to standing and lying times, bout durations, and number of bouts (Ledgerwood et al., 2010). Final datasets included total hourly standing and lying times and total daily standing and lying times, number of bouts, and bout durations.

On some occasions, a logger fell off or was misplaced on the calf. In these cases, data from the last normal observation of logger placement to the time point of the abnormal observation were excluded. During the wildfire events, data were excluded from only 1 calf due to a dropped logger. Additionally, during events when anything other than normally scheduled management and husbandry duties were performed on or within the vicinity of the calves, including all sample collections and animal handling, data were excluded to avoid confounding events that may have resulted in altered activity. This resulted in the removal of the same 184 h of data for each calf across a total of 2,290 recorded hours per calf, which included 18 out of 117 h when PM_2.5_ was above 35 µg/m^3^.

All statistical procedures were conducted in SAS version 9.4 (SAS Institute Inc., Cary, NC). Distribution of residuals was assessed for normality visually and using Kolmogorov-Smirnov tests. Possible correlations between PM_2.5_ and THI, as well as between hPM_2.5_ and hTHI, were analyzed using PROC CORR. Potential bias in coefficient estimates and SEM due to multicollinearity between PM_2.5_ and THI, and between hPM_2.5_ and hTHI, was explored using PROC REG. Multicollinearity is a problem when tolerance (**TOL**) values are <0.1 (Schreiber-Gregory and Jackson, 2017), variance inflation factors (**VIF**) are >10 (Myers, 1990), and condition indices (**COLLIN**) are >5 (Belsley et al., 1980). Based on these cut-off values, multicollinearity did not affect coefficient estimates or SEM in our study.

Daily activity data were analyzed as total minutes per day spent engaging in that behavior using general linear mixed models (PROC MIXED) with daily PM_2.5_ and THI as fixed effects and day within calf ID as a repeated effect. Hourly activity data were analyzed as percentages using generalized linear mixed models with a β distribution (PROC GLIMMIX) with hPM_2.5_ and hTHI as fixed effects and hour within calf ID as a random effect.

To assess effects of novelty of exposure to wildfire PM_2.5_ on calf behavior, PM_2.5_ concentrations 24 h before (baseline) and the initial 24 h of the 2 smoke events (event 1 and event 2) were analyzed using general linear models (PROC GLM), with day as a fixed effect (d 1 = 24 h before event 1, d 2 = initial 24 h of event 1, d 3 = 24 h before event 2, d 4 = initial 24 h of event 2). Tukey-Kramer tests were used for post hoc assessments of differences between PM_2.5_ concentrations across the days. The percentages of time spent standing and lying during the 24 h before the wildfire event and during the first 24 h of each wildfire event were assessed with mixed models (PROC MIXED). Additionally, to evaluate the degree of behavioral alterations before and during each wildfire event, the difference between the percentage of time spent lying or standing during each wildfire event was subtracted from the percentage of time spent standing or lying before each wildfire event. These data were assessed with mixed models using the PROC MIXED procedure. Calf was included as a random effect and day as a fixed effect. These data are presented as LSM ± SEM.

There were 2 wildfire events that resulted in daily average PM_2.5_ concentrations up to 113.5 µg/m^3^ and hPM_2.5_ up to 150.0 µg/m^3^ (Figure 1). Daily average THI reached 65.4 and hourly THI 69.9 during the smoke events (Figure 1). Average calf age during the 2 events was 55 and 62 d of age, respectively. Daily average PM_2.5_ and THI and hPM_2.5_ and hTHI were slightly negatively correlated (−0.12 and −0.07, respectively, both *P* < 0.0001). However, multicollinearity did not contribute to bias in coefficient estimates or SEM (TOL >0.99; VIF <1.02; COLLIN <2.50).

**Figure 1:**
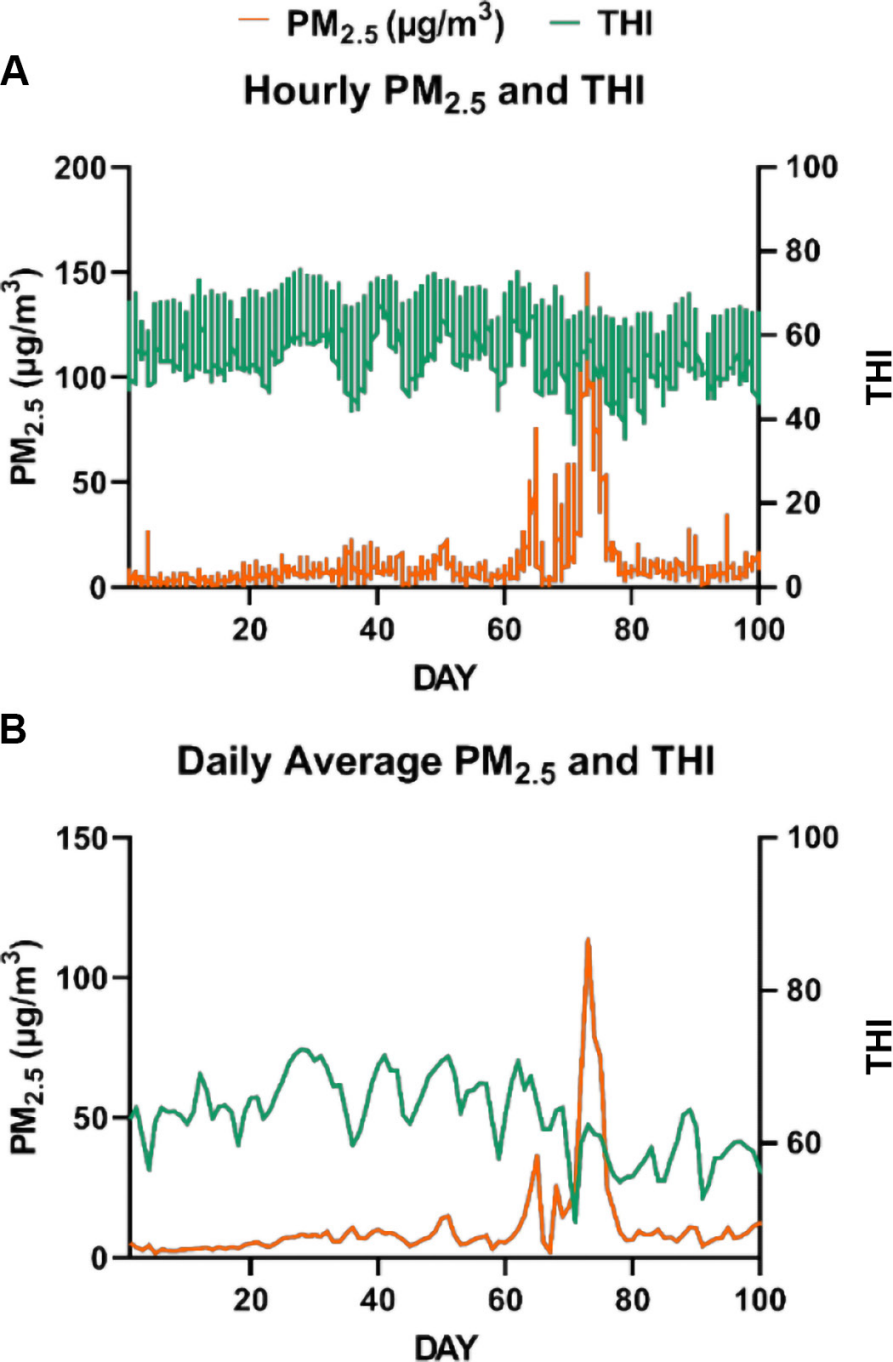
Ambient fine particulate matter (PM_2.5_) concentrations and temperature-humidity index (THI) through the study period of July through October 2022. (A) Hourly PM_2.5_ and THI, (B) daily average PM_2.5_ and THI.

Elevated wildfire PM_2.5_ concentrations decreased daily standing time and bout duration (Table 1, both *P* < 0.05), and nonsignificantly increased the number of daily standing bouts (*P* = 0.09). Daily lying time was increased as a result of higher PM_2.5_ (*P* < 0.001). No change was observed in daily lying bout duration or number of bouts in relation to elevated PM_2.5_. Contrarily, percentage of time spent standing hourly was increased with greater hPM_2.5_ (Table 1; *P* < 0.0001). Additionally, percentage of time spent lying hourly was decreased with higher hPM_2.5_ (*P* < 0.0001). The THI also had an effect on several behavioral variables. For instance, higher THI nonsignificantly increased daily lying bouts while decreasing daily standing bout duration (*P* = 0.06 and *P* < 0.01, respectively). Further, increased hTHI decreased hourly percentage of time standing, while increasing percentage of hourly lying time (both *P* < 0.0001).

**Table 1:** Relationships between daily behavior variables (standing and lying time, bout duration, and bouts), temperature-humidity index (THI), and wildfire-derived particulate matter (PM_2.5_) and hourly behavioral variables (percentage time spent standing and lying hourly), hourly PM_2.5_ (hPM_2.5_), and hourly THI (hTHI)^1^

Model predictor		Variable					
		Standing time	Standing duration	Standing bouts	Lying time	Lying bout duration	Lying bouts
Daily							
Intercept							
	β	338.59	40.15	15.23	1,101.40	67.66	9.56
	SEM	65.5	5.57	3.02	65.50	14.02	6.62
	*P*	—	—	—	—	—	—
PM_2.5_							
	β	−1.02	−0.06	0.02	1.02	−0.004	0.003
	SEM	0.27	0.03	0.02	0.27	0.07	0.03
	*P*	0.0002	0.03	0.09	0.0002	0.96	0.92
THI							
	β	0.72	−0.27	0.03	−0.72	−0.22	0.20
	SEM	1.03	0.09	0.05	1.03	0.22	0.11
	*P*	0.49	0.002	0.53	0.49	0.33	0.06
Hourly							
Intercept							
	β	0.09	—	—	−0.09	—	—
	SEM	0.11	—	—	0.11	—	—
	*P*	—			—	—	—
hPM_2.5_							
	β	0.002	—	—	−0.002	—	—
	SEM	0.0004	—	—	0.0004	—	—
	*P*	<0.0001	—	—	<0.0001	—	—
hTHI							
	β	−0.02	—	—	0.02	—	—
	SEM	0.001	—	—	0.001	—	—
	*P*	<0.0001	—	—	<0.0001	—	—

1 Holstein heifer calves (n = 13) were monitored to 90 d of age. Daily data were analyzed using mixed models, with day within calf as a random effect and PM_2.5_ and THI as fixed effects. Hourly data were analyzed using generalized linear mixed models with a β distribution, with hour within calf as a random effect and PM_2.5_ and THI as fixed effects. Data presented are estimates (β), SEM, and *P*-values.

To analyze responses to novel exposure, the behavior of calves during the first 24 h of the 2 separate wildfire events was assessed. Each wildfire event resulted in an increase in PM_2.5_ concentrations from baseline (i.e., the 24 h before each event; event 1: during = 35.1 µg/m^3^ vs. baseline = 25.1 µg/m^3^, *P* < 0.0001; event 2: during = 72.8 µg/m^3^ vs. baseline = 25.1 µg/m^3^, *P* < 0.0001). Baseline PM_2.5_ concentrations for each event were similar (*P* = 1.0), but PM_2.5_ concentrations during both events were different, with the first event producing lower average daily PM_2.5_ concentrations than the second (*P* < 0.0001). For the first 24 h of each wildfire event, there was a reduction in percentage of time standing (*P* < 0.0001) and an increase in percentage of time lying (*P* < 0.0001) compared with baseline (Figure 2). Behavior differed between the 2 baselines and during the events; percentage of time standing was greater and percentage of time lying was lower in event 2 compared with event 1 (all *P* ≤ 0.0001). The degree of behavioral change for each event also differed between event 1 and event 2, with a larger change in percentage of time spent standing and lying from baseline to during event 1 as compared with event 2 (standing time, event 1: 4.3 ± 0.55% vs. event 2: 3.6 ± 0.55%, *P* = 0.0001; lying time, event 1: 4.3 ± 0.54% vs. event 2: 3.6 ± 0.54%, *P* < 0.0001).

**Figure 2:**
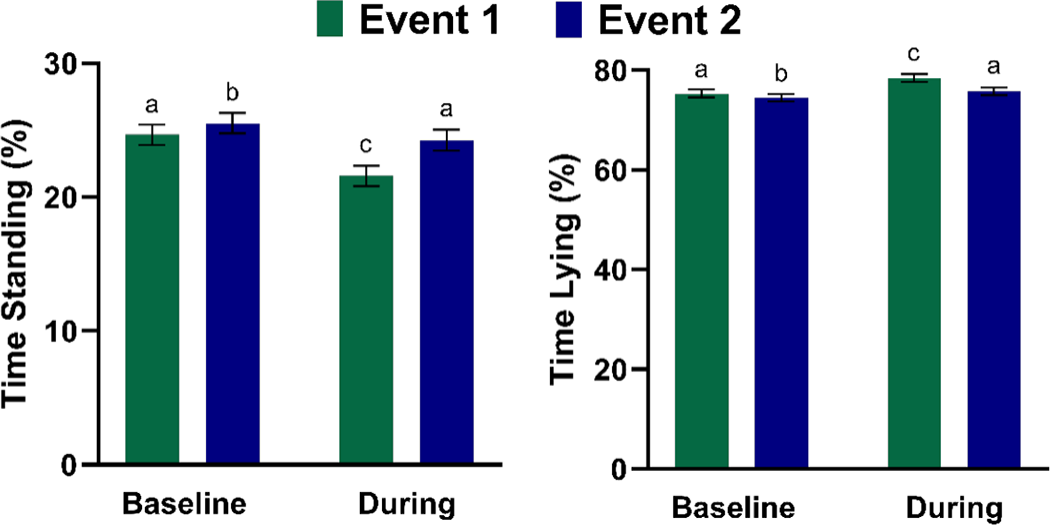
Relationship between percentage of time standing and lying in the 24 h before (baseline) and initial 24 h (during) of 2 separate wildfire events (event 1 and event 2). Holstein heifer calves (n = 13) born in July 2022 were monitored through 90 d of age. Disparate letters indicate significant differences at *P* < 0.05. Error bars denote SEM.

Wildfires produce smoke that afflicts high-producing regions in the US dairy industry. The present study investigated dairy calf behavioral responses to wildfire PM_2.5_ exposure. On days with increased wildfire PM_2.5_, calves spent less time standing and more time lying per day and calves had a nonsignificant increase in standing frequency with shorter standing bout durations, indicating that wildfire PM_2.5_ affects calf activity. Contrarily, exposure to higher hPM_2.5_ resulted in greater percentage of time spent standing and reduced percentage of time spent lying per hour. The opposing hourly compared with daily behavior response to wildfire PM_2.5_ suggests that daily behavioral data do not adequately capture behavioral alterations that occur with more rapid fluctuations in PM_2.5_ (hours vs. days). Rather, daily behavioral patterns reflect responses to PM_2.5_ across longer time scales. Indeed, our group has previously reported persistent physiological responses in cows and calves that occur for several days following wildfire PM_2.5_ exposure (Anderson et al., 2022; Pace et al., 2023).

Although limited, previous research on wildfire smoke effects on animal behavior has been conducted in a few nondomesticated species (Black et al., 2017; Stawski et al., 2017; Erb et al., 2018). Wild animals in fire-prone regions experience selection pressure for their ability to successfully respond to warning signs of fire, such as the sight and smell of smoke (Nimmo et al., 2021). Thus, behavioral responses to fire appear to involve adaptations that promote survival (Kreling et al., 2021). However, dairy cattle are managed in systems that may limit behavioral expression, as has been observed in calves. For example, calves spend more time standing per day and perform more locomotor play behavior when given double the space of a conventional hutch (Jensen and Kyhn, 2000; Hulbert et al., 2019). At present, it is unclear whether behavioral adaptations to wildfires have been evolutionarily conserved in domesticated cattle, and if these responses may be modified by confinement and space restriction.

Herein, we explored calf responses to wildfire smoke, a novel sensory stimuli, and found that calves exhibited greater behavioral responses with exposure to the first wildfire smoke event compared with the second event, even though the first event produced lower PM_2.5_ concentrations. Calf behavior is, at least to some extent, governed by visual and olfactory cues, which calves use to explore their environment (Adamczyk et al., 2015). However, previous research suggests that cattle become habituated to olfactory cues (Wilson et al., 2002). Further research is needed to determine if calves become habituated to sensory cues in wildfire smoke.

The behavioral alterations observed in the present study may be associated with physiology and health. Calves experience inflammation and respiratory irritation with greater combined wildfire PM_2.5_ and THI (Pace et al., 2023), and malaise may affect behavior. Behavioral changes can be important mechanisms enabling stress adaptation and survival (McEwen, 1998). For example, resting more during an immune challenge can enable energy allocation toward physiological pathways that enhance survival (Hart, 1988). Concomitant changes in energy metabolism and behavior have been previously described; orangutans exposed to wildfire smoke rest more, yet produce greater urine ketones, which is indicative of a shift in energy metabolism, and possibly negative energy balance (Erb et al., 2018). We reported similar changes in energy metabolism, including greater circulating ketones, in preweaning calves following combined wildfire PM_2.5_ and THI increases (Pace et al., 2023). Thus, it is possible that calf behavior during wildfire events are linked responses that involve energy allocation, discomfort, mounting of an immune response, or a stress response.

To our knowledge, this is the first study evaluating the behavioral responses of calves to novel increases in hazardous air pollutants from wildfires. Our results indicate that calf behavior is affected by wildfire PM_2.5_ exposure and that responses elicited are dampened across subsequent exposures. It is worth noting that wildfire smoke contains a heterogeneous mixture of gaseous and particulate pollutants, so there may be other cues, in addition to PM_2.5_, that calves are detecting and responding to. Future research is warranted to elucidate proximate mechanisms contributing to attenuated responses to subsequent wildfire smoke exposures as well as addressing links between various smoke “cues,” behavioral patterns, and calf health, welfare, and productivity. This work will continue to contribute to the dairy industry's resilience to a changing climate with greater wildfire activity.
